# Nasal anatomy and sniffing in respiration and olfaction of wild and domestic animals

**DOI:** 10.3389/fvets.2023.1172140

**Published:** 2023-07-14

**Authors:** Jinxiang Xi, Xiuhua April Si, Mauro Malvè

**Affiliations:** ^1^Department of Biomedical Engineering, University of Massachusetts, Lowell, MA, United States; ^2^Department of Mechanical Engineering, California Baptist University, Riverside, CA, United States; ^3^Department of Engineering, Public University of Navarre, Pamplona, Spain; ^4^Biomedical Research Networking Center in Bioengineering, Biomaterials and Nanomedicine (CIBER-BBN), Madrid, Spain

**Keywords:** maxilloturbinate, ethmoturbinate, lab animals, livestock, nose function, animal models, COVID-19

## Abstract

Animals have been widely utilized as surrogate models for humans in exposure testing, infectious disease experiments, and immunology studies. However, respiratory diseases affect both humans and animals. These disorders can spontaneously affect wild and domestic animals, impacting their quality and quantity of life. The origin of such responses can primarily be traced back to the pathogens deposited in the respiratory tract. There is a lack of understanding of the transport and deposition of respirable particulate matter (bio-aerosols or viruses) in either wild or domestic animals. Moreover, local dosimetry is more relevant than the total or regionally averaged doses in assessing exposure risks or therapeutic outcomes. An accurate prediction of the total and local dosimetry is the crucial first step to quantifying the dose-response relationship, which in turn necessitates detailed knowledge of animals’ respiratory tract and flow/aerosol dynamics within it. In this review, we examined the nasal anatomy and physiology (i.e., structure-function relationship) of different animals, including the dog, rat, rabbit, deer, rhombus monkey, cat, and other domestic and wild animals. Special attention was paid to the similarities and differences in the vestibular, respiratory, and olfactory regions among different species. The ventilation airflow and behaviors of inhaled aerosols were described as pertinent to the animals’ mechanisms for ventilation modulation and olfaction enhancement. In particular, sniffing, a breathing maneuver that animals often practice enhancing olfaction, was examined in detail in different animals. Animal models used in COVID-19 research were discussed. The advances and challenges of using numerical modeling in place of animal studies were discussed. The application of this technique in animals is relevant for bidirectional improvements in animal and human health.

## 1. Introduction

### 1.1. Overview of animal nasal anatomy and functions

The animal nose (nasus) is a complex structure that plays a critical role in many animals’ respiratory, olfactory, and thermoregulatory systems. Its anatomy and functions vary significantly among species, reflecting adaptations to different environments and lifestyles (1). Understanding the anatomy and functions of the animal nose can provide valuable insights into the overall health and well-being of these animals.

The nasal cavity is divided into two main parts: the external nares and the internal nasal cavities. The external nares are the openings in the animal’s face through which air enters the nose. The internal nasal cavities are located within the head and consist of a complex network of airways, mucous membranes, and tissues that perform various functions. The internal nasal cavities are lined with mucous membranes, which contain specialized glands that secrete mucus that humidifies, filters, and warms the inhaled air. Convoluted structures called turbinates often feature the internal cavity. The turbinates can be further divided into two major parts: maxillomoturbinate (MT) and the more complicated ethmoidal turbinate (ET), with the MT responsible for respiration and the ET for olfaction. The olfactory epithelium is located in ET and contains olfactory receptor neurons that can detect and process odors and transmit the processed information to the brain.

### 1.2. Essential role of animal models in veterinary science and other disciplines

Animals have been extensively used as surrogate models for human health studies, as well as in veterinary science (2–5). They have been instrumental in advancing our understanding of various diseases and conditions in veterinary medicine (6–8). They are used in various stages of research, from basic studies of disease mechanisms to testing the efficacy and safety of new treatments or drugs. The most common animal models used in veterinary science include rodents (e.g., mice and rats), domestic animals (e.g., cats, dogs, horses, livestock), and non-human primates. Each species has its own unique characteristics and benefits, making them suitable for certain types of research. Challenges also exist in extrapolating test data between different species, especially from animal data to humans, because animal anatomy, physiology, and genetics differ from humans to varying degrees. As a result, physiology-based modeling has emerged as an alternative tool in the past decades, mainly due to the advent of imaging technologies and increasing computational power, which make it practical to consider the myriad of influential factors that were otherwise prohibitive in the past.

There are other reasons that discourage the usage of animal models and promote physiology-based modeling, including ethical concerns, high cost, time-consuming, difficulty in controlling variables, and high variability in results. The use of animals in research raises ethical and moral concerns about the treatment and welfare of the animals. Maintaining animals and cultivating disease models in animals can be expensive, let alone that such procedures often take a long time, delaying the development of new treatments and therapies for human patients. In some cases, it can be challenging to control variables such as diet, environment, and social interaction, which can impact the results of a study. Moreover, different strains of animals can respond differently to the same treatment or intervention, making it difficult to obtain consistent results. On the other hand, developing a species-specific physiology-based model for a specific disease can greatly meliorate the above setbacks, given the developed model had been validated for its accurate embodiment of the fundamental underlying factors.

In this review, we examined the nasal anatomy and physiology of different animals, including dog, rat, rabbit, deer, rhombus monkey, cat, and other domestic and wild animals. Special attention was paid to the similarities and differences in the vestibular, respiratory, and olfactory regions among different species. The ventilation airflow and behaviors of inhaled aerosols were described as pertinent to the animals’ mechanisms for ventilation modulation and olfaction enhancement. In particular, sniffing, a breathing maneuver that animals often practice enhancing olfaction, was examined in detail in different animals. The implications of airflow and aerosol deposition in animal toxicology studies and inhalation drug delivery were also presented. Animal models used in COVID-19 research were discussed. The advances and challenges of using numerical modeling in place of animal studies were discussed. The application of this technique in animals is bidirectional in animal and human health: the knowledge obtained using animal models can be applied to improve veterinary medicine and animal life while not human medicine only.

## 2. Diversity in shapes and functions of animal noses (nares)

### 2.1. Nasal anatomy and functions

The anatomy of an animal’s nose can vary in size, shape, and structure depending on its habitat, diet, and behaviors. This diversity can be attributed to differences in the functions of their noses, such as flow regulation, warming/moistening airflow, filtering particles, and detecting odors (9). For example, some species have long, narrow nostrils for detecting scents, while others have large nostrils for breathing in hot environments. Additionally, some animals have highly developed olfactory systems and specialized structures for detecting scents, such as the vomeronasal organ in snakes, while others have limited or no sense of smell (10). Equipped with an exquisitely tuned sensory system, animals predominately use their nose to communicate with each other, including perceiving danger, locating food, attracting mates, and demarcating territories.

Various techniques have been utilized to investigate the nasal structure of diverse mammalian species, including fixed tissue dissections, airway casting, as well as medical imaging modalities such as magnetic resonance imaging (MRI) or computed tomography (CT) scans (11–13).

### 2.2. Allometric scaling of animal respiration

**Figure 1:**
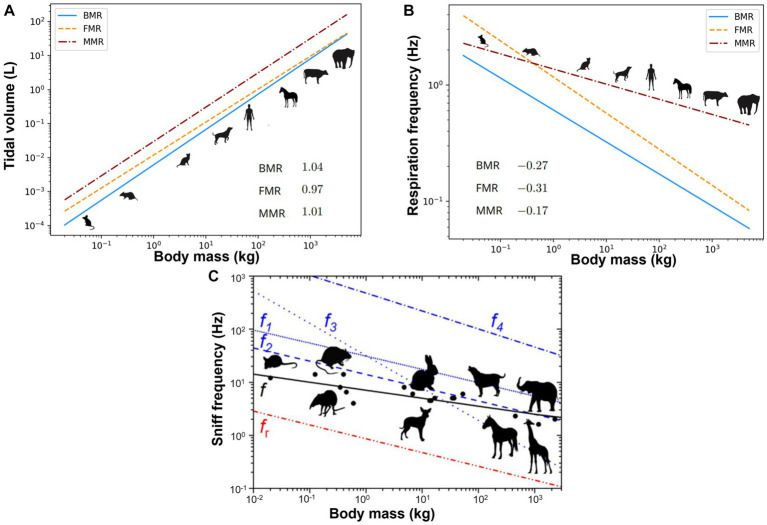
Allometric plots vs. animal body mass: **(A)** tidal volume (15), **(B)** respiration frequency (15), and **(C)** sniff frequency (16). BMR, basal metabolic rate; FMR, field metabolic rate; MMR, maximal metabolic rate.

Species with varying body masses can differ significantly in their nasal airway size, breathing frequency, and tidal volume. Allometry is the study of the relationship between the size and shape of biological structures. It is a crucial tool for understanding the biology of animals and how it evolves. The allometric scaling of respiration can be described by power law relationships, where the rate of respiration parameter (*R*) is proportional to body mass (*M*) raised to a power (*b*): *R* = *kM^b^*, where *k* is a constant (14). Figures 1A,B show the allometric scaling for the tidal volume and frequency, respectively for animals with a wide range of body weight (15). For most mammals, the value of *b* for the tidal volume is typically between 0.97 and 1.04, indicating an approximately linear increase with the body size (15).

### 2.3. Sniffing

Sniffing refers to a tout of rhythmic forced inhalation that enables quick detection of airborne odors (17). It often involves a fast nose waggling at a frequency much higher than the normal breathing frequency. Figure 1C shows the association between the sniff frequency vs. body mass for animals ranging from mice to elephants (16). The best-fit regression for the sniff frequency revealed a coefficient *k* being 8.0 and exponent *b* being −0.18 (black solid line in Figure 1C), reflecting the higher sniff rates in smaller animals (16). Studies showed that sniffing was coupled to olfactory neural responses, synchronizing the sniff cycle with brain information encoding for odor identity or concentration (18). Animals adjust sniffing as a function of odorants. Their olfactory mucosa has a similar function to a chromatograph that differentiates odorants based on absorptive properties, and they can modulate sniffing behaviors to manipulate airflow and direct odorant molecules to specific olfactory sites. Rats can detect high-sorption chemicals more easily than low-sorption chemicals. However, sniffing at a lower frequency with a higher flow rate better detected low-sorption chemicals (19). A fast sniff can detect an odor quickly, but a too-fast sniff can significantly reduce the odor concentration, lowering the signal amplitude to the noise level (16).

Craven et al. (20) measured the canine sniffing characteristics in dogs ranging from 6.8 to 52.9 kg. A sniffing frequency of 5 ± 2 Hz with sinusoidal waveforms was observed regardless of the dog breeds or masses. This was different from the tidal volume, which scaled allometrically with body mass (14, 21). Similar sniffing frequencies (5.33 + 0.7 Hz) were also measured by Crawford (22). They also found that the canine respiratory tract had a resonant frequency of 5.28 + 0.3 Hz and speculated a minimal sniffing energy expenditure at such frequencies. By comparison, humans sniff at a much lower frequency, i.e., 0.3–0.7 Hz (23). Interestingly, the sniff frequency of dogs coincides with the olfactory neural theta frequency, corroborating the notation of sniffing-coding synchronization between the nose and brain (24, 25). The active sniffing in response to an odor stimulus lasted from 0.5 s to 2 s with a series of consecutive sniffs. It often started from a weak sniff, then increased its amplitude towards the peak and gradually decreased afterward.

Youngentob et al. (26) quantitatively analyzed sniffing characteristics in rats and suggested the sniffing behavior (amplitude and frequency) could be different for different odors and for different concentrations of the same odor. The rat’s sniffing started with one or two inhalations and was followed by alternating inhalations and exhalations, with the peak sniffs near the end. It was suggested that a twelve-parameter response could capture the complexity of the sniffing patterns in rodents in responses to various odorant stimuli by quantifying the temporal and volumetric aspects of sniffing behavior (27). Among them, seven parameters were associated with inhalation (7: amplitude, duration, frequency, peak flow rate, mean flow rate, time to reach the peak, volume during the first 0.5 s), three with exhalation (3: peak flow rate, mean flow rate, time to reach peak), while the reaming two were inhalation-to-exhalation ratio and inter-sniff interval (28, 29). The average inhalation volume during the first 0.5 s following odorant onset represented an informative metric since it reflected the initial olfactory response to odorant stimuli. Walker et al. (30) measured the respiration duration in conscious Sprague–Dawley rats using a plethysmograph. Results showed that at low breathing frequencies (*f*), the expiration:inhalation ration (E:I) >1, while it reduced to one when *f* > 2.5 Hz (i.e., exceeding 150 breaths per minute).

Wesson et al. (31) measured the mouse sniffing pattern by means of intranasal transient pressure. The respiration frequency in quiescent mice was 3–5 Hz, which was higher than in rats. With odor stimuli, the sniff frequency rose to 12 Hz or so and exhibited swift fluctuations in waveform, amplitude, and frequency. There was significant variation in the sniffing behavior observed across different tasks, as well as within different behavioral phases of each task.

Freeman et al. (32) investigated the rabbit’s ability to learn and respond to different odors by measuring the sniffing frequency in 21 New Zealand white rabbits. By using statistical analysis of digitized pneumograph recordings, the sniffing episodes were non-invasively identified amidst the respiratory activity in the background. The rabbits were presented with different odors, and their sniffing behaviors were recorded and analyzed. It was observed that rabbits could adjust their sniffing frequencies depending on various odor stimuli or olfactory cues. In a familiar environment, a basal rate of exploratory sniffing (5.6–6 Hz) existed, which increased sharply upon new stimuli and could remain high with continuous stimuli reinforcement. When the odor stimuli diminished, the sniff frequency first experienced a steep decline before gradually approaching the basal rate, a phenomenon commonly found in macrosmatic animals (33).

## 3. Physiology-based modeling of respiration and olfaction

Nasal anatomy and physiology of different species were presented in an order based on (1) the year and impact of the studies, (2) lab animals, livestock, wild animals, and (3) land animals vs. bats/fishes/birds. Considering the diverse nose functions among species, varying levels of detail were presented for each species.

### 3.1. Dog (*Canis familiaris*) olfaction and biomimetic design

**Figure 2:**
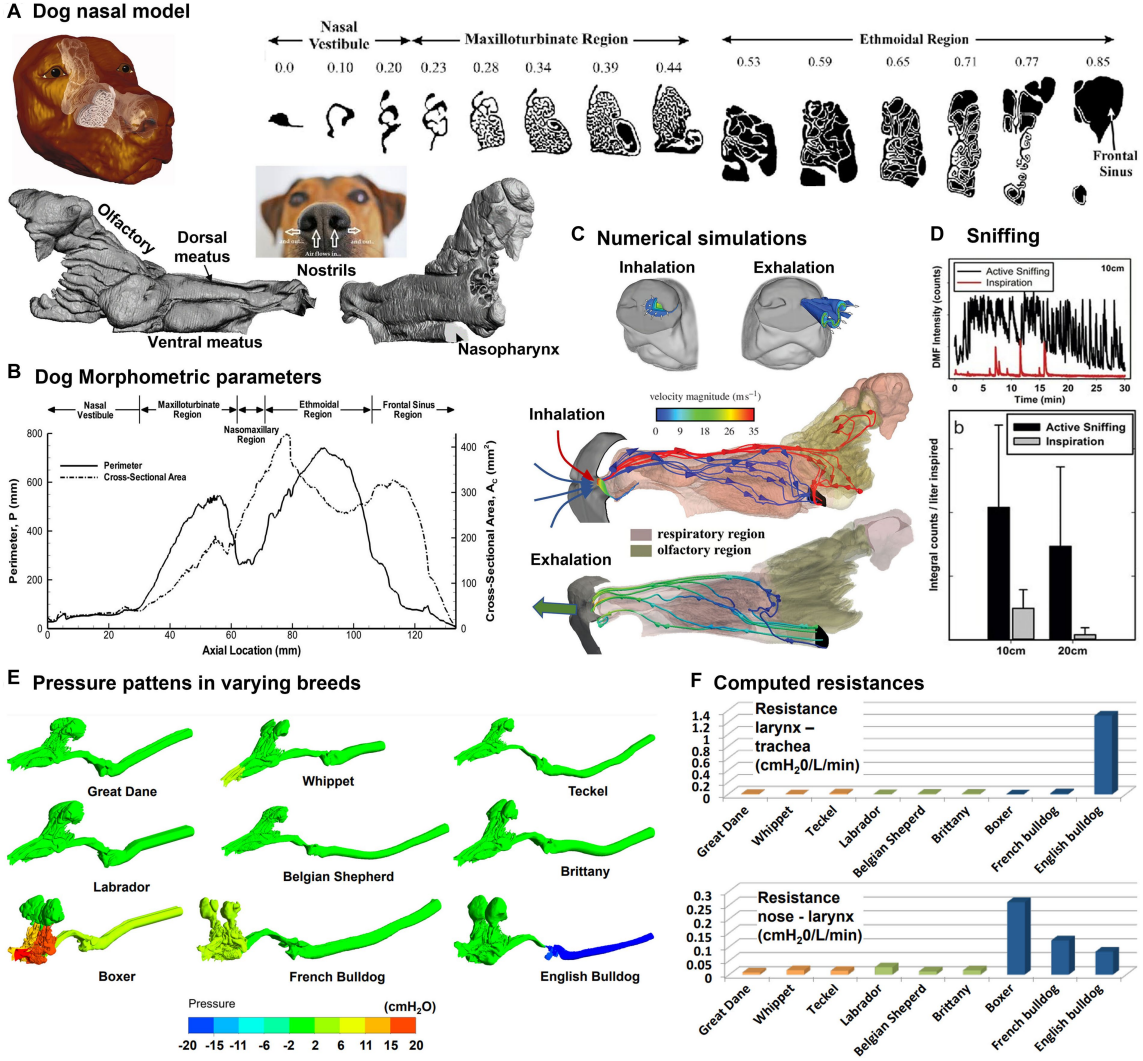
Canine nasal airway model: **(A)** MRI images along the axial direction and reconstructed nose model with three regions: vestibule, maxilloturbinate, and ethmoturbinate (39); **(B)** morphometric parameters (perimeter and cross-sectional area of the sagittal slices), **(C)** numerical simulations of inspiratory and expiratory flows, **(D)** sniffing (40), **(E)** comparison of the pressure patterns between different breeds (Dolicho-, Meso-and Brachycephalic nasal airway and trachea models), and **(F)** computed resistances at different airway locations (41).

Dogs come in a variety of breeds and have the largest variation in body size of all terrestrial vertebrates (34). Dogs have often been selected as surrogate models for humans in inhalation and lung function tests (35–37). A recent review of canine olfaction can be found in Kokocińska-Kusiak et al. (38), which surveyed the physiological mechanisms and anatomical features that are implicated in the process of detecting and identifying odors. Pioneering modeling and simulation studies on canine nasal morphology were conducted by Craven and colleagues in 2007 (39), who developed a detailed nasal airway model based on high-resolution MR scans, which might have been the first time that the intricate fine structures of the nasal conchae were exhibited (Figure 2A, left panel). The branching maxilloturbinate and double-scroll shaped ethmoturbinate appear structurally distinct, which underlie their functions in respiration and olfaction, respectively (Figure 2A, right panel). Morphometric parameters were also quantified and Figure 2B shows the cross-sectional area and perimeter of the coronal slices vs. the axial direction from the naris. A series of computational fluid dynamics (CFD) simulations have been conducted of the fluid dynamics in the canine nose with different aims, from human to veterinary medicine: with an emphasis on model verification (42), for animal health improvement, computing flow resistance in English Bulldogs (43), comparing anatomies and resistances in Dolicho-, Meso-and Brachycephalic breeds (41), or analysing the clinical outcomes of rhinoplasty in French Bulldogs (44, 45), for studying sniffing (20, 40), and odor transport (46) (Figure 2C). Airflows near the naris can be very different between inhalation and exhalation, with a small hemispherical zone (1 cm diameter) upstream of the naris during inhalation vs. a jet flow during exhalation (Figure 2C). The hemispherical zone corresponds to the distance that has observed between the odor source and the dog nose while tracking scents (47). In particular, the sniffing frequency was measured in dogs ranging between 6.8–52.9 kg, which was found to be ±2 Hz with a sinusoidal waveform regardless of the dog breeds and masses (20) (Figure 2D, upper panel). Sniffing was predicted to dispense 2.5 times more airflow to the olfactory region, resulting in 2.5 to 3 times more absorption of odorants that are highly-and moderately-soluble in the olfactory mucosa (40) (Figure 2D, lower panel). Different brachycephalic dogs present a wide variability airflow resistance, despite the lack of respiratory signs. The anatomy in apparent healthy brachycephalic breed has been found to promote non-uniform pressure patterns (Figure 2E) and considerable higher flow resistances in comparison with Dolicho-and Mesocephalic breed (41) (Figure 2F). Furthermore, the simple resection of the nares and of the soft palate may not be sufficient to correct the basic problem in brachycephalic breeds (44, 45). The clinical indication of the increase of resistance is the respiratory distress or decreased airflow due to prolonged inspiratory time that results in an increased effort of breathing (48).

Due to their olfactory acuity and close relationship with humans, many interesting interactions between dogs and humans have been observed, some of which may have meaningful implications for human health. Dogs can discern and identify the scent of a particular individual from a group of persons up to 48 h after the scent has been created and even in the presence of other stronger odors (49). By using their sense of smell, dogs can readily distinguish human emotions like fear or happiness (50). Trained dogs can accurately detect seizures (51), narcolepsy (52), diabetes (53), and malaria parasites (54). In addition, dogs underwent training for cancer detection in expiratory breaths, urines, feces, and biopsy samples with different diseases: large intestine (55), bladder (56), prostate (57), lung (58), ovary (59), and breast (60). Without any training, dogs were able to detect the development of melanoma in their owners (61). Furthermore, the dogs demonstrated the ability to detect not only melanoma that was developing on the skin of the patient, but also cancer cells that were deliberately placed on the skin of healthy individuals (61). Trained dogs have also been used to detect diseases in other animals, such as bovine respiratory disease (62), as well as bioaerosols and explosives (63, 64).

### 3.2. Sprague–Dawley rat and scaled models

**Figure 3:**
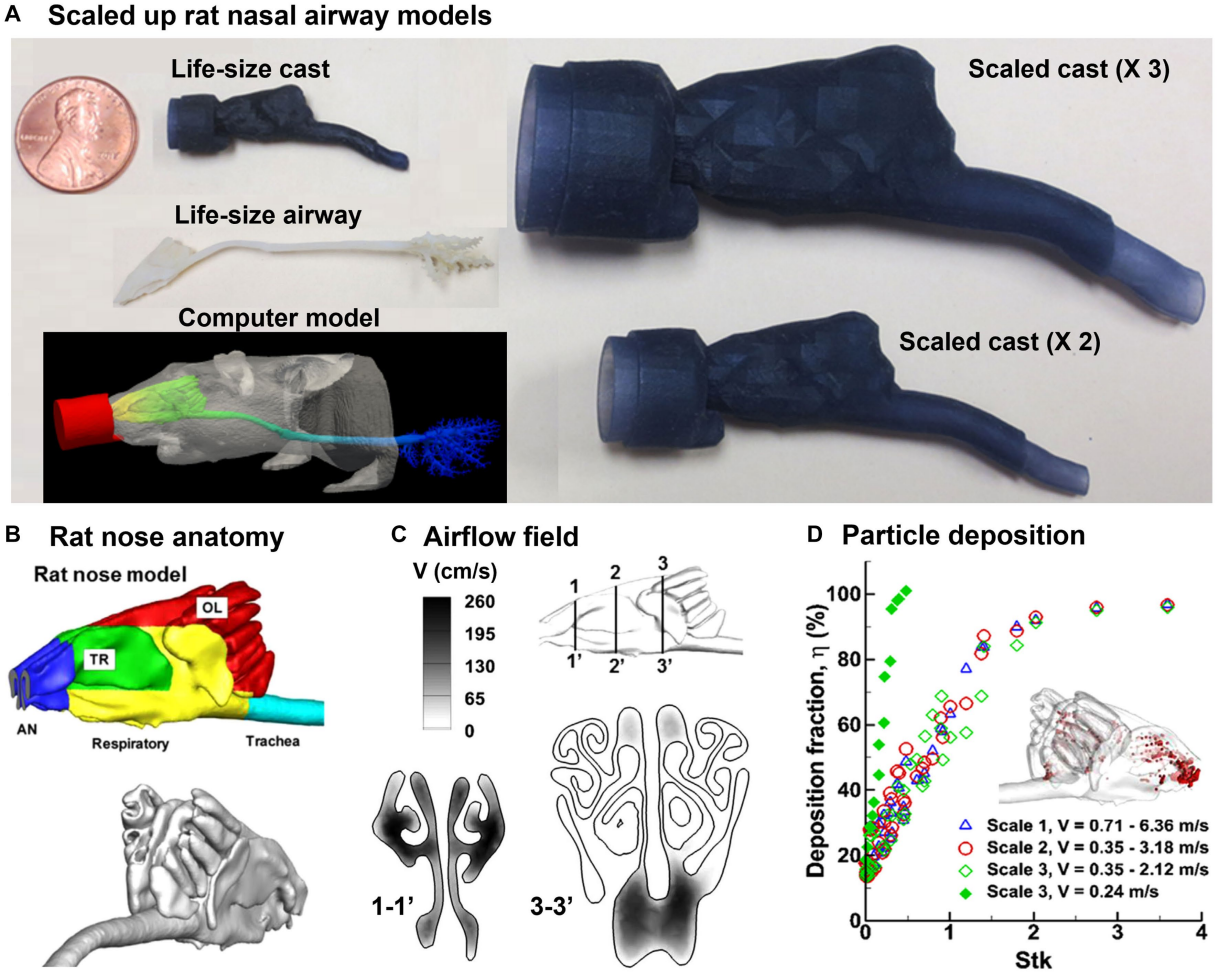
Nasal airway model of a Sprague–Dawley rat (80): **(A)** life-size and scaled rat nose models of a Sprague–Dawley rat compared to a penny, **(B)** rat nose and anatomy and different functional regions, **(C)** numerically predicted airflow field, and **(D)** precited deposition fraction vs. stokes number (St_k_).

The Sprague–Dawley rat is a commonly used laboratory animal in scientific research, such as toxicology, pharmacology, and behavioral research. Its distinctive moist and highly vascularized nose makes it well-suited for respiratory and olfactory studies (65–68). Schroeter et al. (69–71) proposed a pharmacokinetic-driven CFD model in the Sprague–Dawley rat nose model and predicted the nasal uptake of inhaled hydrogen sulfide. Similarly, Corley et al. (72) simulated acrolein deposition and subsequent pharmacokinetics (PK) in both rats and humans. Rats were used to evaluate the risks of exposure to highly reactive and soluble vapors like formaldehyde (73–79). Overall, it was observed that the vapor uptake in a specific region was influenced by several factors, including the airway anatomy, flow rate, vapor concentration, tissue thickness, metabolism rate, and partition coefficient between air and tissue. In addition, a fraction of volatile vapors would escape the nasal mucosa absorption, which entered the lung and affected lower airways. Clinical evidence has shown high relevance between site-specific deposition and carcinogenesis. However, it is highly challenging to conduct *in vitro* deposition tests using 3D-printed nasal airway casts because of the small size of the rodent nasal cavity. Figure 3A shows a life-size nasal airway model of an adult Sprague–Dawley rat in comparison to a penny coin. Its complex nasal geometry and small size make both handling and measurement difficult.

It is of interest to know whether scaled-up rodent nasal models can be used for physiologically equivalent deposition studies (Figure 3A). Kolanjiyil et al. (81) estimated and compared the total and regional particle deposition in mouse versus human lung, using upper airway lung models based on morphometric data with the aim of comparing the retention and clearance kinetics between species. Xi et al. (80) evaluated the feasibility of scaled-up rodent nasal models for 0.5–24 μm aerosols by scaling up the nasal geometry by two and three times (i.e., scale 2 and scale 3), respectively. In doing so, an image-based rat nasal model was reconstructed from MRI scans of a male, 10 weeks-old Sprague Dawley rat with a weight of 0.3 kg (72), which comprised five sections: vestibule (blue color), turbinate (green), respiratory zone (yellow), olfactory region (red), and trachea (blue), as displayed in Figure 3B. Results showed that the equivalent airflow dynamics and dosimetry could be achieved when the scaled rodent models had the same Reynolds number and Stokes number, and when *Fr* > 50 (Figures 3C,D). Scaled models offer several benefits, including facilitating the preparation and handling of the airway replica casts of small animals, as well as enabling regional dosimetry quantifications in these casts. Tremendous differences were also revealed between rodents and humans in nasal anatomy, physiology, and olfactory area, which led to large differences in the total and regional dosimetry between rodents and humans. Knowledge of the level of confidence in using scaled rat models to approximate human inhalation dosimetry will facilitate the design of animal tests, comparison in dosimetry between rats and humans, and outcome extrapolation from rat to humans (82, 83).

### 3.3. New Zealand white rabbit: respiration, sniffing, and olfaction

#### 3.3.1. Rabbit nasal airway development and characterization

**Figure 4:**
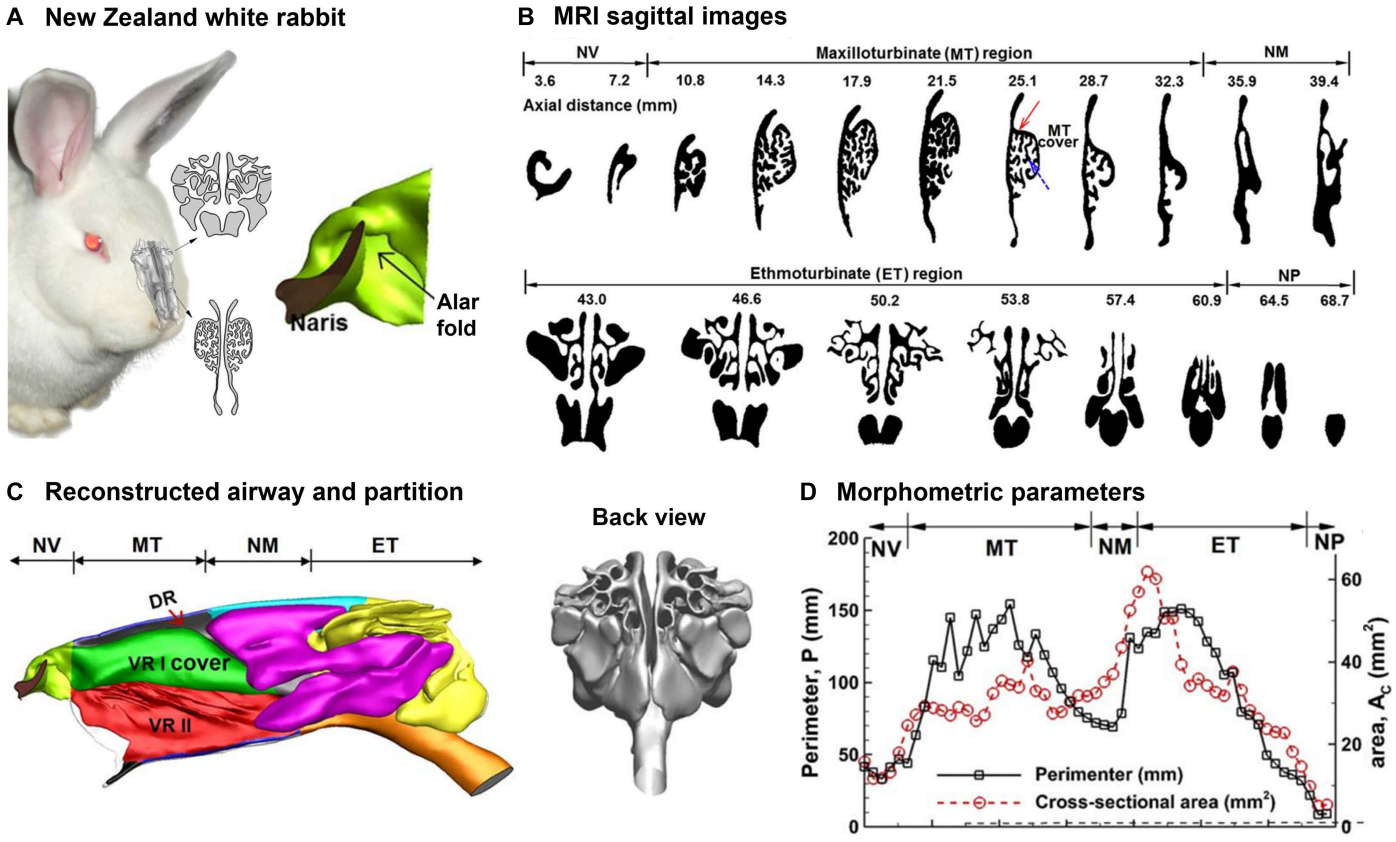
Nasal airway model of a New Zealand white rabbit (89): **(A)** 3D printed and computer model, **(B)** sagittal MRI images from the nostril to the trachea, **(C)** reconstructed rabbit nasal airway geometry with different functional regions, and **(D)** morphometric parameters (perimeter and cross-sectional area of the sagittal slices).

Rabbits (*Oryctolagus cuniculus*) have highly sensitive noses and use sniffing as a means of gathering information about their environment at home or in the wild (84, 85). The nasal airway structure of NZW rabbits exhibits a greater degree of complexity than those of humans or monkeys, while it shares more resemblances with the nasal airways of other macrosmatic animals like rats and dogs (39, 72, 86–88). Figure 4A shows the 3D printed and computer nasal airway model of an adult NZW rabbit. One unique feature was the spiral-shaped vestibule, as illustrated to the right of Figure 4A, where three channels were ramified from the comma-shaped nostril. The lower point of the base of the nostril is linked to the inferior maxilloturbinate (green line), whereas the apical point is linked to the dorsal maxilloturbinate (red line). The sagittal MRI scans of the rabbit nose are depicted in Figure 4B at different axial positions, which were acquired from a rabbit cadaver using a 2.0-tesla MRI scanner and an acquisition resolution of 512 × 512 (90). Negus (91) categorized the turbinate (or concha) of mammals into four types: folded, single scroll, double scroll, and branching. Of these, the branching structure was deemed the most advanced and providing the largest surface area. The nose was separated into two cavities by the septal wall and each cavity was further divided into four functional sections: nasal vestibule, maxiloturbinate (MT), nasomaxillary (NM), and ethmoturbinate (ET), as shown in Figure 4C. The MT can be subdivided into three parts, namely the dorsal respiratory (DR) zone, the ventral respiratory I (VR I) zone which features a folded cover, and the ventral respiratory II (VR II) zone. In terms of function, the DR zone provides a direct pathway to the ET as a shortcut to the olfactory mucosa. In the rabbit nose, the VR I zone boasts the most intricate architecture and is responsible for both air-conditioning and distribution of inhaled air. This zone can direct the air either to the ET for odorant sensing or to the trachea for breathing. Situated at the ventral end of the MT and aligned with the trachea, the VR II zone is responsible for warming and moistening inhaled air before directing it toward the lungs. A back view of the ET is presented in Figure 4C (right panel), which exhibits a scroll-like architecture.

The rabbit nasal airway dimensions were quantified for each sagittal slice. Figure 4D shows the perimeter and area of each slice vs. the distance from the naris. There are two crests in the cross-sectional dimension (*A*_c_ and *P*) that, respectively, correspond to the maxilloturbinate (MT) and ethmoturbinate (ET). In comparison, the cross-sectional dimensions (*A*_c_ and *P*) within the nasal vestibule are relatively modest. Moreover, the *P* − *A*_c_ ratio is greater in MT than ET, which suggests a higher degree of MT structural geometric intricacy than MT (Figure 4B, upper vs. lower panel).

#### 3.3.2. Numerical simulations of rabbit respiration and olfaction

Several numerical studies have been conducted on rabbit respiration and olfaction. A mathematical deposition model for NZW rabbits was created by Asgharian et al. (92) to investigate inhalation anthrax. However, this model had a restricted scope to fine and coarse particles only. This was because there was insufficient data available on nanoparticle deposition tests. The inhalation dosimetry of anthrax was numerically analyzed by Kabilan et al. (93), revealing that the deposition distribution was highly sensitive to local flows and aerosol size. An integrated experimental-computational approach was proposed by Hess et al. (94), which involved the use of *in vitro* data from rabbits to construct a physiologically based biokinetic model (PBBK). This PBBK could be linked to an existing aerosol dosimetry model, enabling consideration of species-specific variability. More recently, Xi et al. (89, 95) numerically studied the anatomical effects on rabbit breathing, air conditioning, olfaction, as well as the sniffing effects on nanoparticle deposition, which were explained in more detail.

**Figure 5:**
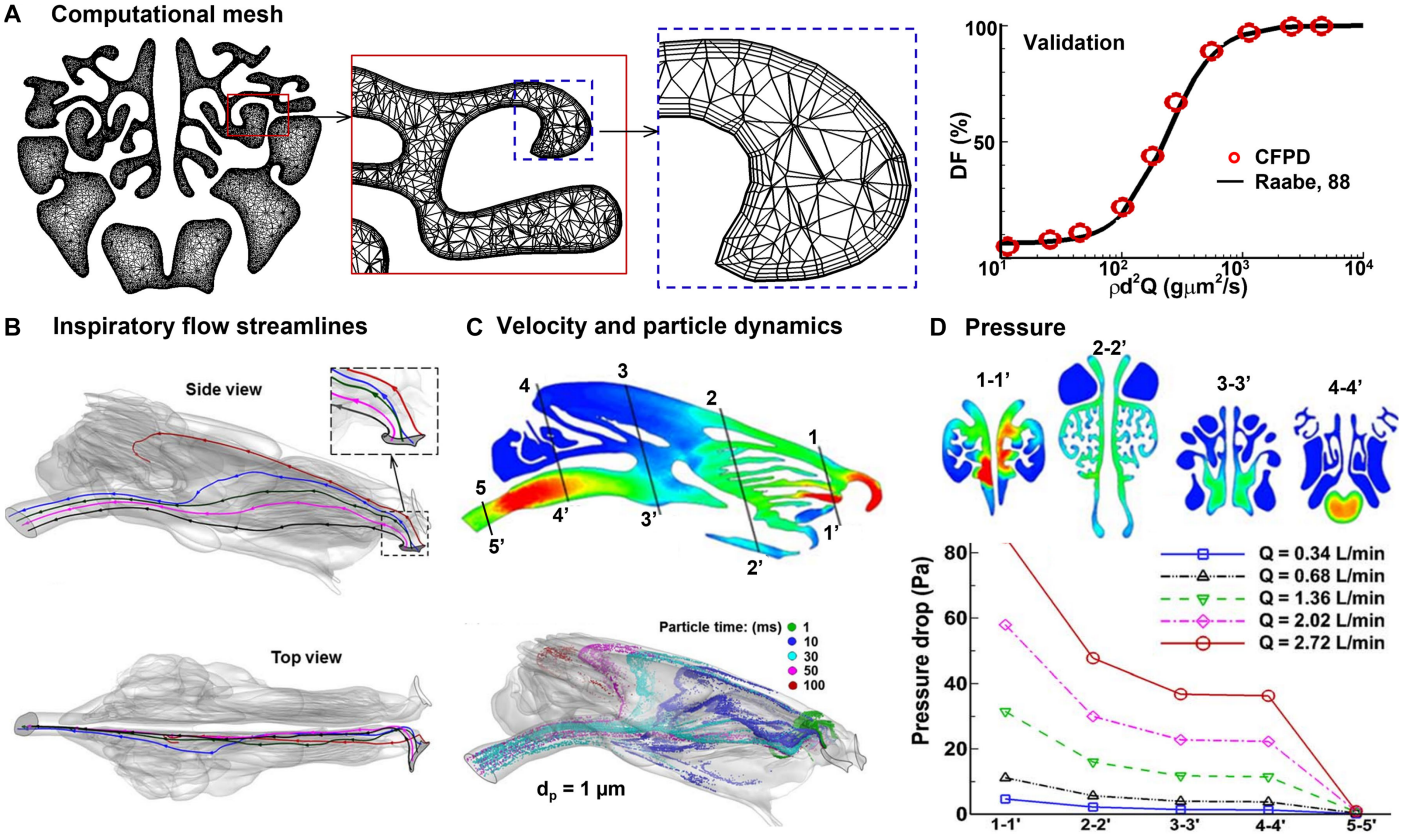
Numerical simulations of rabbit respiration (89, 95): **(A)** computational mesh with body-fitted prismatic cells and experimental validation, **(B)** inspiratory airflow streamlines, **(C)** inspiratory velocity and particle dynamics, and **(D)** inhalation resistance.

A high-quality computational mesh is required for accurate numerical simulations. Figure 5A displays the computational mesh within the MT at three distinct scales, namely global (coarse), local (fine), and near-wall (ultrafine). Within the near-wall region, there exist four layers of prismatic cells, with the first layer cell at the height of 15 μm. Model validation was conducted in two steps: to determine the optimal mesh density, a mesh sensitivity analysis was performed using various mesh densities. The analysis began with a mesh size of 1.1 million and subsequently increased incrementally, reaching mesh densities of 1.8, 2.6, 3.6, and 4.9 million. The results were considered grid-independent when the change was smaller than 0.5%. Based on these findings, a final mesh size of approximately 3.6 million cells was selected for this study. Secondly, the numerically predicted deposition of micrometer particles at normal breathing was compared to experimental deposition data in rabbits (2). The high degree of agreement between the measured and simulated dosimetry of inertia particles, coupled with the verification studies for nanoparticles (96), provided assurance in the numerical methodology employed in this study (Figure 5A, right panel).

Simulation results of rabbit respiration and olfaction are shown in Figures 5B–D. In Figure 5B, the streamlines originating from the nostril tip are found to travel toward the olfactory region (highlighted in red). On the other hand, the streamlines originating from the middle nostril go into the MT (colored in blue, green, and pink), while those from the nostril base enter the inferior meatus (depicted in black). As per the findings of Corley et al. (90), only an insignificant fraction (1%) of the inspiratory flow goes into the ET. Given the fact that olfactory neurons are highly sensitive and fragile, this low flow rate could be sufficient to sense the entrained chemicals, while also safeguarding the neurons from potential harm by environmental toxins. Second, the vestibule has a distinctive design that facilitates the distribution of inhaled air. This is achieved through the two spiral curves from the ala fold, with the second curve being partitioned into two channels by the dorsal concha. One channel directs airflow to the middle meatus, while the other channel leads to the dorsal meatus, ultimately culminating in the olfactory recess (Figures 4A, 5B). This was the first time to demonstrate that the spiral-shaped vestibule was critical in distributing the inhaled air into respiration and olfaction. When the air was inhaled *via* the spiral-shaped vestibule, the airflow and particles twisted from the nearly level slit-inlet to the perpendicular nasal valve (rightmost panel, Figure 5B). It was also shown that sniffing can regulate the flow partition between respiration and olfaction. The distribution of inhaled airflow in the rabbit nose exhibited a significant degree of heterogeneity (Figure 5C) and varied under different breathing conditions. Only a low portion of inhaled particles penetrated the posterior ET. The nasal breathing resistance increased nonlinearly with the respiration flow rate (Figure 5D), indicating enhanced mixing or turbulence occurs at a high inhalation rate of 2.02–2.72 L/min.

#### 3.3.3. Rabbit sniffing and olfaction

**Figure 6:**
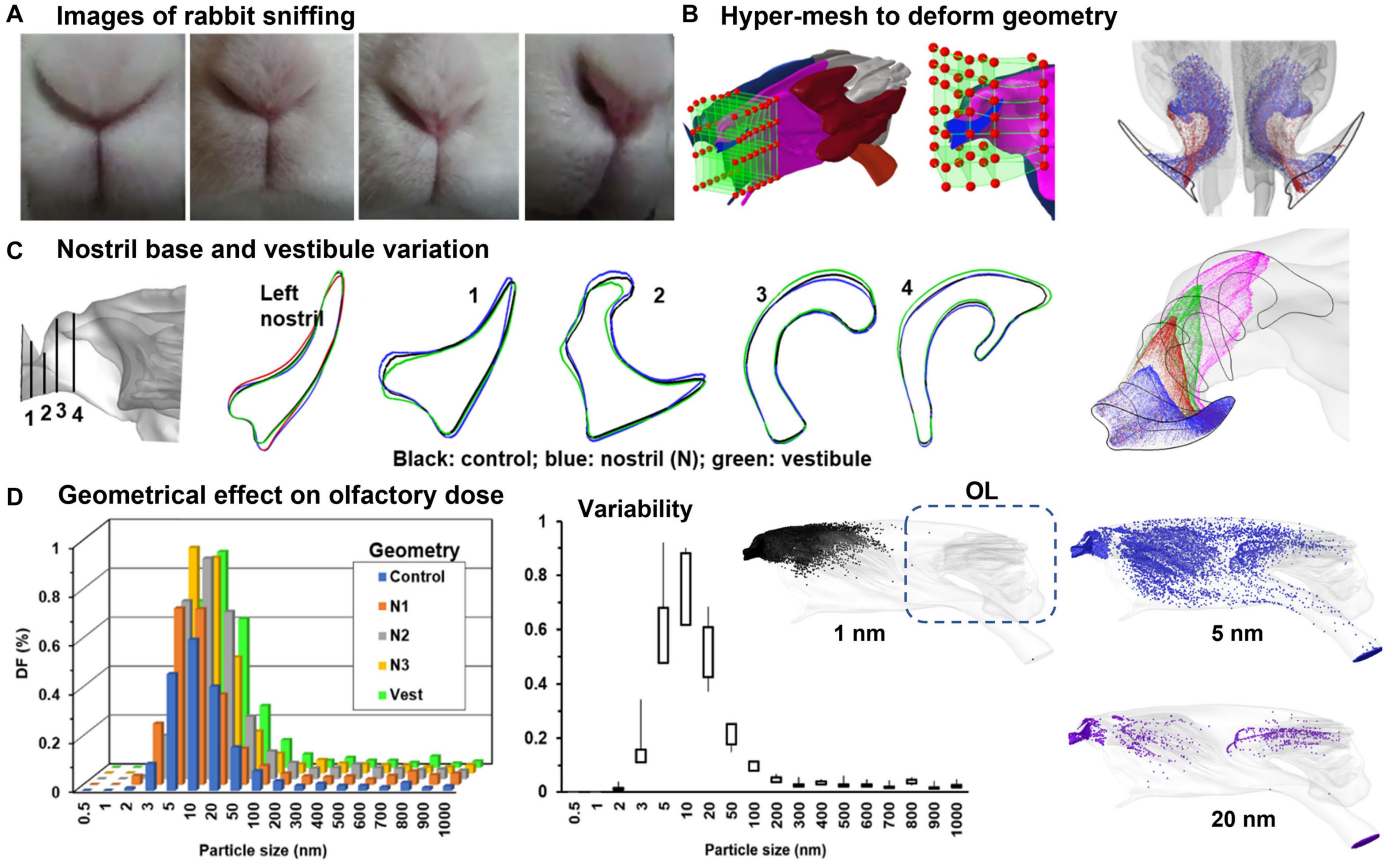
Numerical simulations of rabbit sniffing (95): **(A)** images of rabbit sniffing, **(B)** using hyper-mesh to deform the local geometry with prescribed magnitude, **(C)** deformed front nose with four cross-sectional contours, **(D)** geometrical effect on nanoparticle deposition in the olfactory region.

Informed by high-speed video images of rabbit sniffing, the nose model, referred to as “control,” underwent additional deformations (Figure 6A). While sniffing, the nostrils and vestibule both change shapes. In order to simulate this change, the nostril slit widths at the middle were measured and the maximum width variation was calculated. HyperMorph was then used to progressively expand the left nostril to generate N1, N2, and N3 (Troy, MI, United States) (Figure 6B), with N3 representing the widest nostril. A more detailed description of HyperMorph’s usage can be found in Xi et al. (97–99). The axial profiles of the left nostril in the control group (black) and N3 group (blue) are compared in Figure 6C. Sniffing also causes changes to the vestibule and nasal valve, so a new model that expands the vestibule without significantly altering the nostrils was developed, as denoted “vestibule” with green color in Figure 6C.

To investigate the sniffing effects on olfaction, the olfactory deposition of inhaled nanoparticles was numerically simulated and compared between control and deformed geometries (N1–N3, vestibule) at a sniffing frequency of 6 Hz. Consistently across the control, N1, N2, and N3 groups, enlarging the left nostril enhanced the deposition into the olfactory region (Figure 6D, left panel). The highest variability in the olfactory dosing was predicted for nanoparticles ranging between 5–20 nm (Figure 6D, middle panel). The right panel shows the high sensitivity of the deposition distribution to particle size. For 1 nm particles (black color) with elevated diffusivity, deposition occurred in the front nose only. By contrast, particles with a size of 5 nm exhibited substantial deposition across the entire nasal region, with appreciable doses in the olfactory region (blue color). As particle size increased, the deposition became more confined, resulting in a notable decrease in olfactory doses for particles of 20 nm and larger (Figure 6D).

### 3.4. White-tailed deer, sheep, and pig: complexity and functions

**Figure 7:**
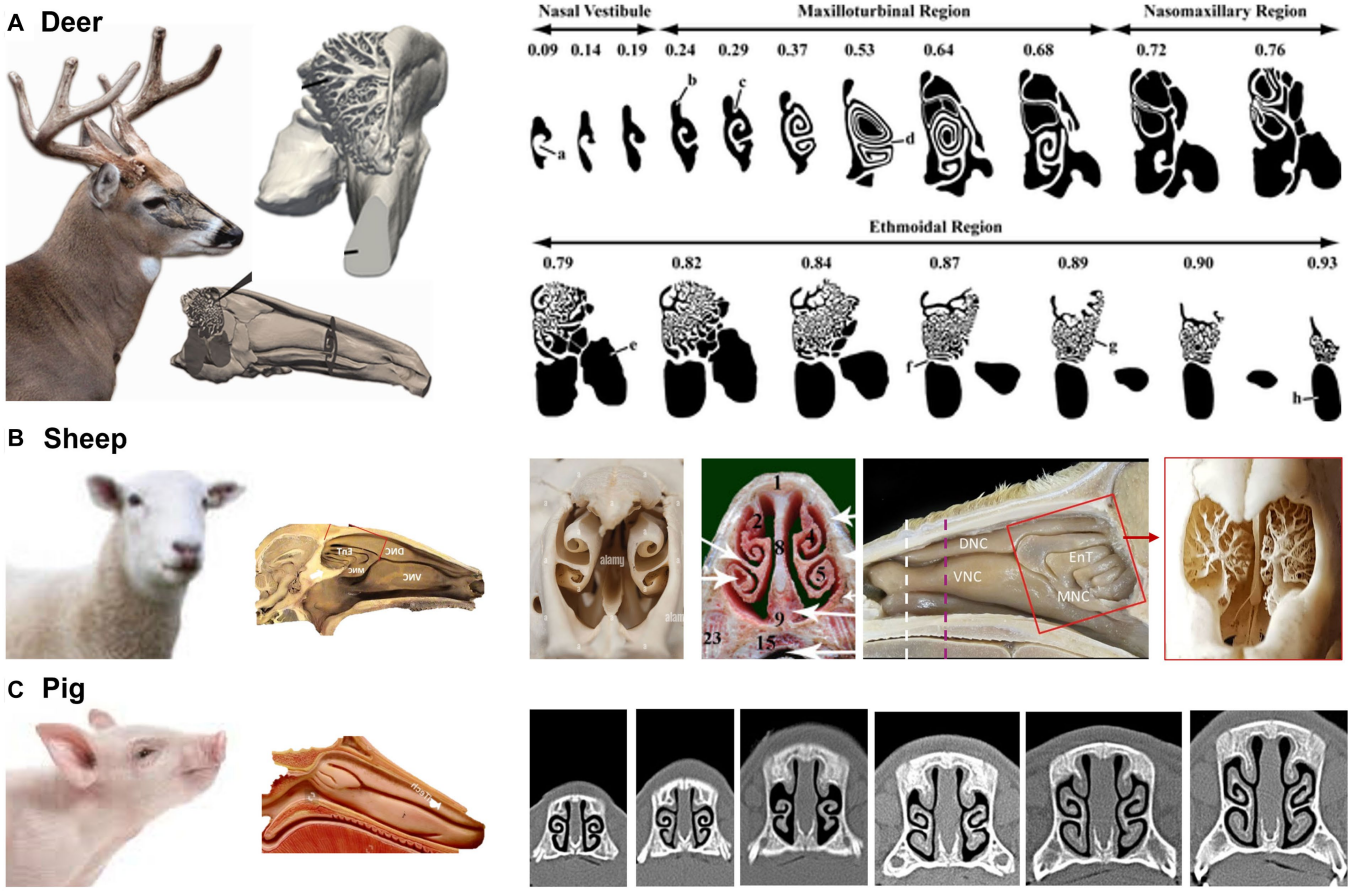
Nasal airway anatomy: **(A)** white-tailed deer (86), **(B)** sheep (100), and **(C)** pig (101).

Ranslow et al. (86) developed a high-resolution MRI-based nasal airway model of an adult white-tailed deer and quantified the nasal morphometric dimensions, including the coronal slice perimeter, cross-sectional area, and nasal wall surface area. Three anatomical features were revealed that facilitate deer respiration and olfaction. As shown in Figure 7A, the nasal structure of white-tailed deer is characterized by a lengthy maxilloturbinate with a double-scroll configuration, which spans about 50% of the nasal fossa and offers the major contact for mass (moisture) and heat transfer. The flow regime within the vestibule and anterior maxilloturbinate can be either transitional or turbulent, with the turbulent mixing boosting the efficiency of heat and moisture exchange. This process can play a significant role in thermoregulation and water conservation in these deer. Second, the deer’s olfactory region features an intricate arrangement of branching ethmoturbinals that differ in their morphology from the single and double-scroll ethmoturbinate observed in other non-primate species (see Table 1). This convoluted folding results in a substantial surface area that facilitates the detection of chemicals within the confined space available for the olfactory function. Thirdly, the dorsal meatus was linked to an olfactory recess, which created unique airflow patterns during sniffing that optimized odor delivery to the olfactory mucosa.

**Table 1:** Comparison of maxilloturbinate (MT) and ethmoturbinate (ET) among different animals.

	MT		ET		Refs.
	Morphology	Complexity	Morphology	Complexity	
Dog	Branching	Higher	Scroll	High	(39, 42, 43)
Rat	Folded	Low	Scroll	High	(67, 68, 76)
Rabbit	Branching	High	Scroll	High	(89, 93, 95)
Deer	Double-scroll	High	Branching	Higher	(86)
Sheep	Scroll	Low	Branching	High	(100)
Pig	Folded	Low	Scroll	Low	(101, 102)
Camel	Double-scroll	Low	Folded	Low	(103, 104)
Cat	Branching	Higher	Scroll	High	(105, 106)
Monkey	Folded	Low	Folded	Low	(72, 107, 108)
Human	Folded	Low	Folded	Low	(109, 110)
Bat	Scroll	High	Scroll	High	(111)

Sheep and deer both belong to the order Artiodactyl (i.e., even number of toes) and are herbivores commonly found in grasslands or forests. As prey animals with similar predators (e.g., wolves), their turbinates also look similar, with scroll-like maxilloturbinate and reef-like (branching) ethmoidal turbinate (100) (Figure 7B). A slight difference in the sheep nose is its concave nasal surface as opposed to the concave nose of the deer.

Pigs have relatively small nostrils compared to sheep. However, pigs have a highly developed sense of smell, which is important for finding food. Pigs use their nose to locate roots, truffles, and other sources of food that are buried in the ground. A pig’s flexible snout is well adapted for rooting in the ground and foraging for food (Figure 7C). Both sheep and pigs have moist nostrils, which help to filter out dust and debris and to keep the airways moist (101). This helps to reduce the risk of respiratory infections. Sheep and pigs both have a keen sense of smell, but pigs have a slightly more developed olfactory system (102). This is due in part to the number and size of olfactory receptors in their nose, which are specialized structures that detect and identify scents.

### 3.5. Horse and camel: breathing resistance, thermoregulation, and water conservation

A racehorse’s nasal airway helps its performance by allowing them to take in large amounts of air quickly and efficiently. The nasal airway is designed to maximize air intake and improve the flow of air to the lungs. The nostrils of a horse are wide and flared, allowing for maximum air intake, and the airways within the nose are straight and unobstructed, reducing resistance and improving airflow, which can improve its endurance and performance during races. Additionally, the horse’s nasal passages are lined with blood vessels, which help to warm and humidify the air before it reaches the lungs, ensuring that the horse’s respiratory system is protected against the harsh conditions it encounters during intense exercise.

**Figure 8:**
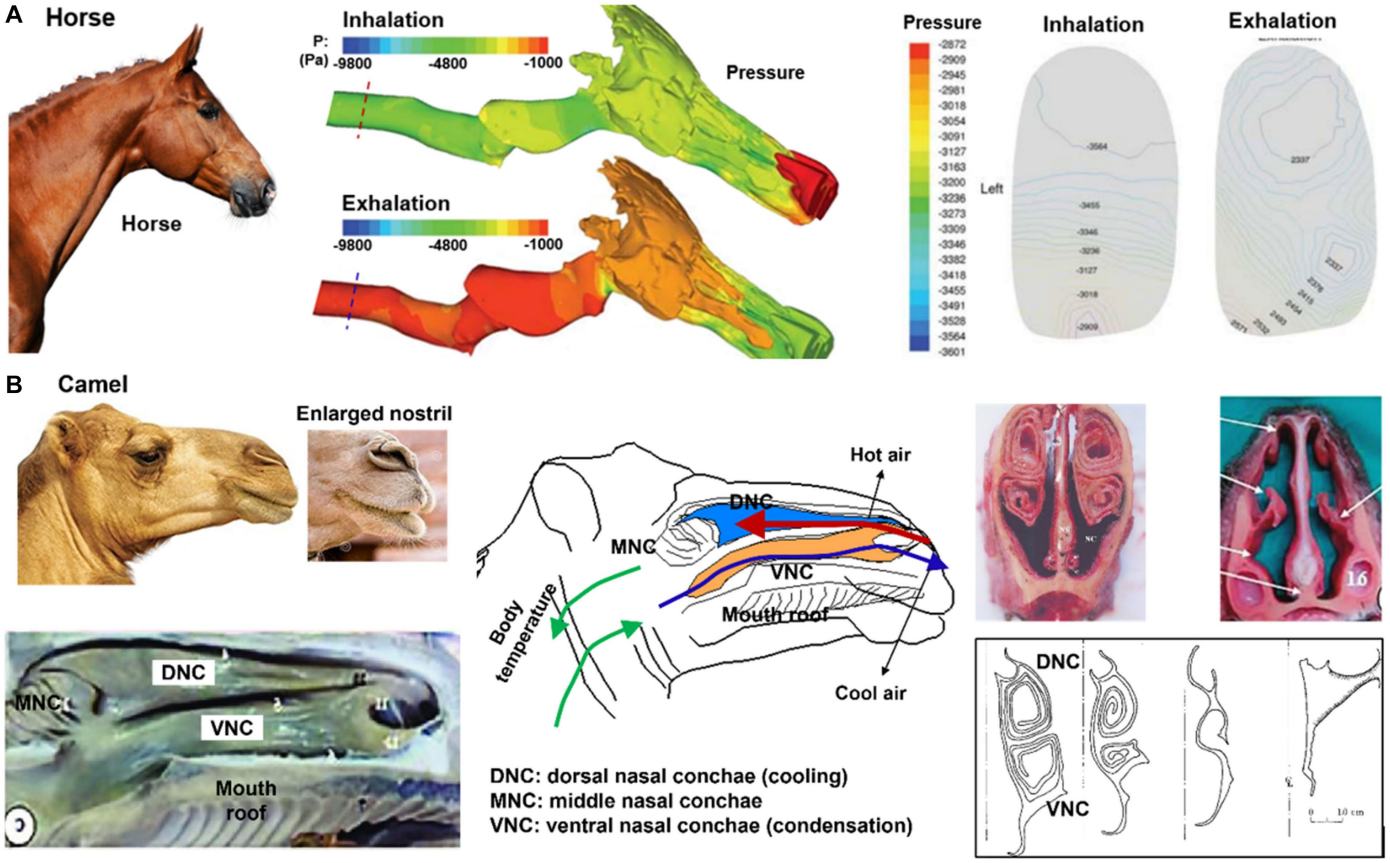
Nasal airway anatomy: **(A)** racehorse (112), **(B)** camel with unique thermal/vapor regulation capacity (103).

Rakesh et al. (112) developed an equine upper airway model from CT scans of a 3 years-old male Thoroughbred racehorse cadaver and simulated the airflow dynamics during exercise using *in-vivo* measured airway pressures as the boundary condition (Figure 8A). This model helped identify regions that were susceptible to dynamic collapses, such as the rostral nasopharynx (pars nasalis pharynges). During inhalation (right panel, Figure 8A), the combination of low pressure and high turbulent kinetic energy caused palatal instability, which was believed to be a significant factor contributing to the high incidence of dorsal displacement of the soft palate (DDSP) in racehorses (112). As a result, considerable muscular activity is needed to support the front part of the nasopharynx during forceful respirations.

For a camel living in a hot and dry environment, its nose plays an important role in thermoregulation and water conservation. Camels are well adapted to life in harsh desert environments and have several physical adaptations that allow them to conserve water and regulate their body temperature. As shown in Figure 8B, camels have long, narrow nostrils that can be closed to prevent the inhalation of sand and dust, and to conserve moisture. The moist lining of the nostrils helps to cool the air that is inhaled, which helps to regulate the camel’s body temperature. Additionally, the shape of the nose helps to humidify the air that is breathed in, which can be important in maintaining respiratory health in dry desert environments (103). The nasal structure also plays a role in water conservation, as the moist lining of the nostrils helps to condense the exhaled moisture and reduce the amount of water lost during exhalation (104). The water conservation is attributed to the nasal mucosa hygroscopic attributes when the camel is dehydrated; the lower vapor pressure on the nasal epithelium absorbs moisture from the exhaled respiratory air, leading to the exhaled air at a relative humidity of less than 100% (113). At the same time, the camel’s nose also has hydrophobic properties, meaning it repels water. This helps to prevent the mucus from becoming saturated with water and allows it to continue to absorb water vapor efficiently. The hydrophobic properties of the camel’s nose are due to the presence of lipids, or fats, in the mucus that helps to create a barrier and prevent water from permeating it (114).

### 3.6. Felidae nasal airway anatomy: cat, bobcat, and cheetah

**Figure 9:**
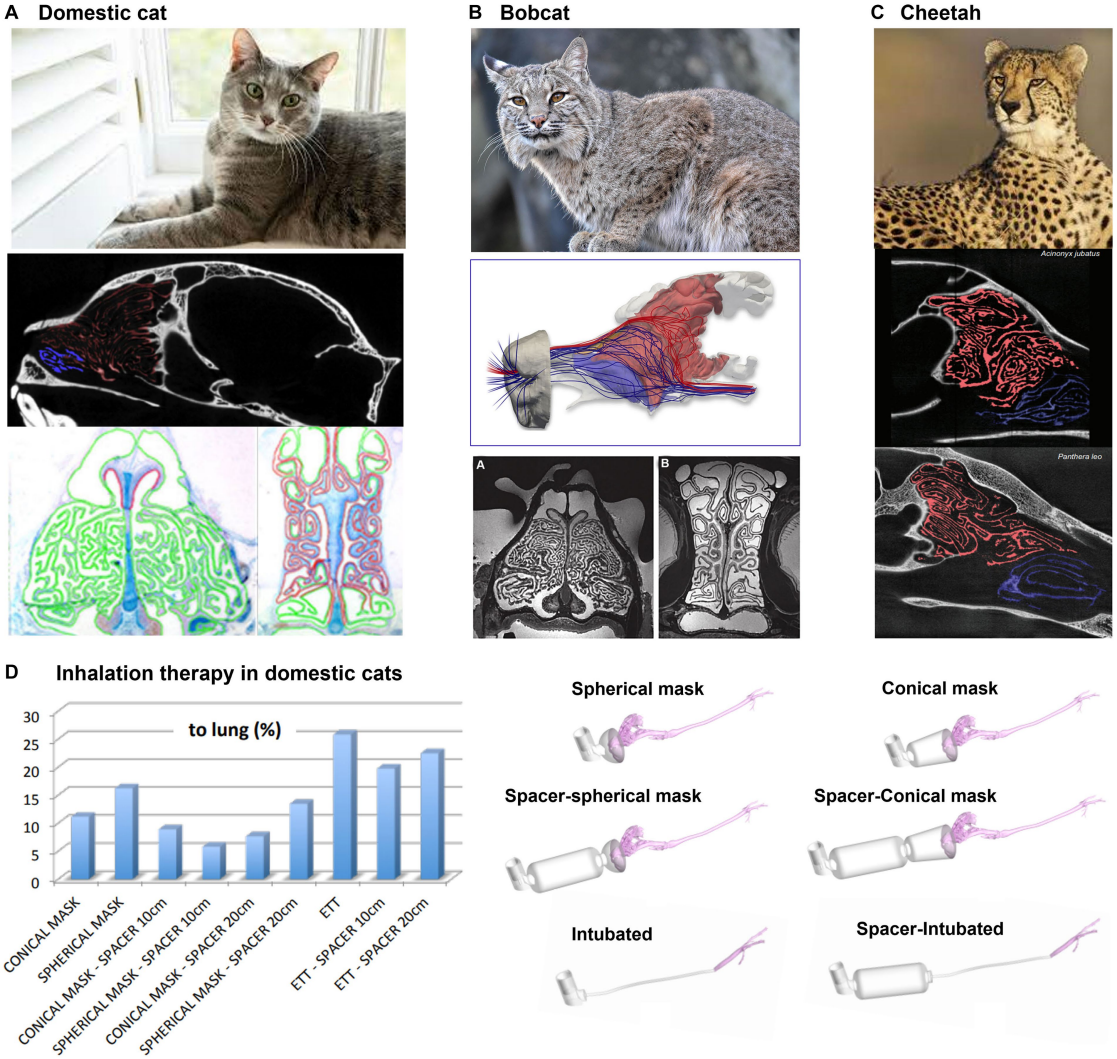
Felidae nasal airway anatomy: **(A)** domestic cat, **(B)** bobcat, **(C)** cheetah, all with highly intricate ethmoturbinate adapted for acute olfaction (105), and **(D)** modeling of inhalation therapy in domestic cats (106).

Cats, bobcats, and cheetahs belong to the same family of Felidae, and thus their ethmoidal turbinates are similar in shape and complexity (105), as shown in Figures 9A–C. They are known for their powerful sense of smell, which they use to locate prey, avoid danger, and communicate with one another. Felids have large and complex ethmoidal turbinates, which gives them a larger surface area for detecting odors (105). This increased surface area helps them to detect a wider range of odors and to identify specific scents more easily (Figures 9A–C). In addition, felids have a unique structure within their nasal cavities called the Jacobson’s organ located near the ethmoidal turbinates (115). It is responsible for analyzing pheromones and other chemical signals. The large surface area of the olfactory tissues, combined with the high number of olfactory receptor neurons, allows felids to detect even the faintest of scents. Additionally, the highly mobile nostrils and naris allow felids to control the flow of air into the nasal cavities, allowing them to optimize their ability to detect odors (116).

In the treatment of feline bronchial disease, the use of a pMDI (pressurized metered-dose inhaler) with a spacer is a common practice for home treatment in asthmatic cats (117). However, in emergency clinical situations where a spacer is not available, pMDI salbutamol is administered to intubated cats either directly through a pre-oxygenation mask or an endotracheal tube (ETT). Using CFD, Fernández-Parra et al. (106) demonstrated that the delivery of salbutamol using an endotracheal tube (ETT) is more effective than using spacer+preoxygenation mask (Figure 9D). In fact, a considerable amount of drug tends to deposit on the muzzle and on the main airways of the animal, independently on the presence or not of a spacer and on its dimension. On the contrary, intubation directly delivers the salbutamol to the trachea. Additionally, the non-cooperative character of cats may even cause a consistent reduction of the percentage of drugs reaching the lung computed *in silico*. Finally, the ventilation conditions, crucial for the drug delivery, for an intubated cat are different with respect to those non-intubated as the animal tends to be stressed during the therapy if not sedated.

### 3.7. Non-human primate: cynomolgus monkey, rhesus monkey and chimpanzee

**Figure 10:**
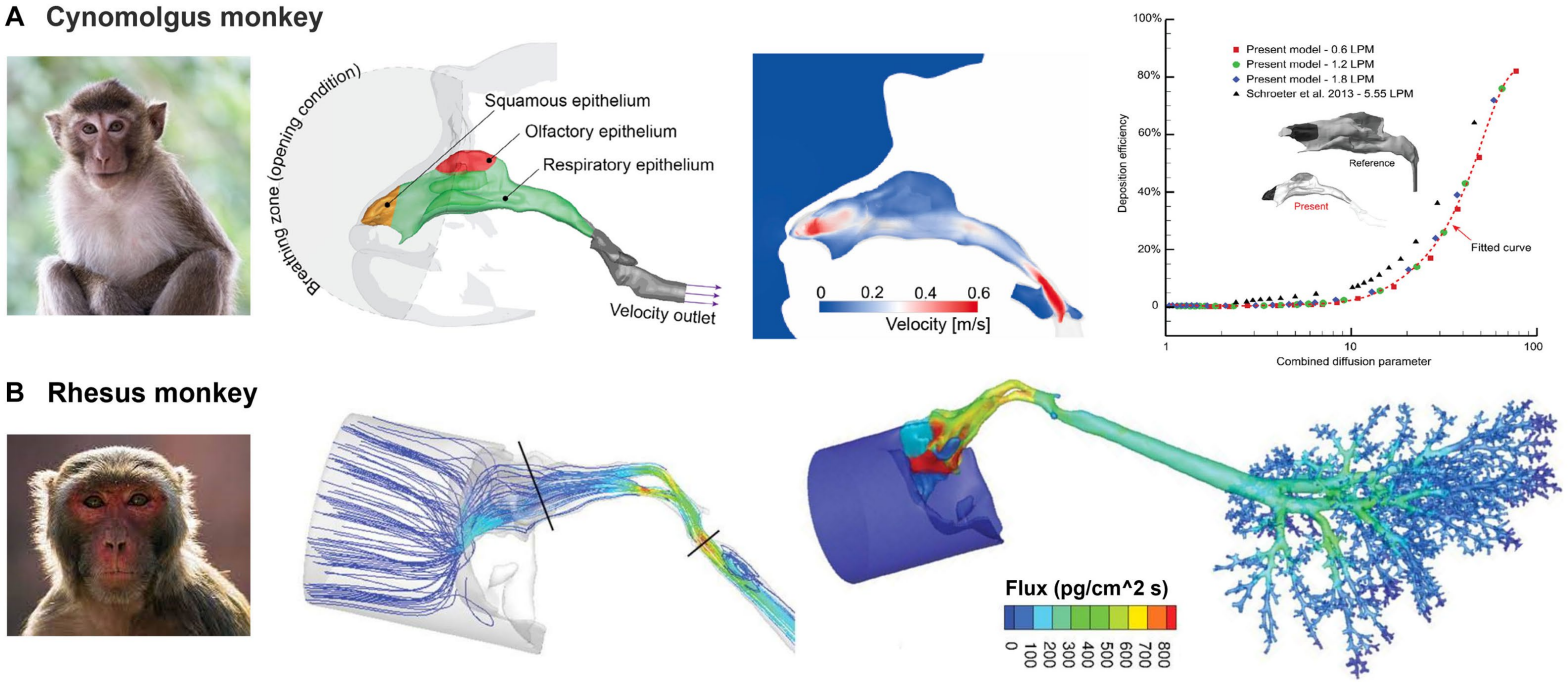
Non-human primate nasal airway models: **(A)** cynomolgus monkey (107), and **(B)** rhesus monkey (72).

Figure 10 shows the nasal airway models for the cynomolgus monkey and rhesus monkey, respectively. They belong to the same family (*Cercopithecidae*), and have some similarities in appearance and behavior, but also have distinctive differences. Cynomolgus monkeys are smaller in size and have distinctive black faces, while rhesus monkeys have red faces and are larger in size. Salguero et al. (118) evaluated rhesus and cynomolgus macaques as potential COVID-19 infection models. Their findings revealed that SARS-CoV-2 could replicate in the upper and lower respiratory tracts of both species and induce pulmonary lesions. Additionally, the immune responses generated against SARS-CoV-2 in these macaques were comparable to those seen in humans with mild COVID-19 infections (118–120).

Compared to dogs and lab animals, the primate nasal airway exhibits a much simpler morphology (Figures 10A,B). By contrast, it is more like the human nose, both of which have curved-up inferior, middle conchae (turbinates). The relative height of the primate nose is smaller than that of humans (109, 110, 121–126), mainly due to the monkey’s forward-protruding face. Dong et al. (107) numerically studied nanoparticle deposition in a cynomolgus monkey nose model and obtained deposition results that were in good agreement with previous studies (Figure 10A). Tian et al. (108) conducted a detailed comparative analysis of the nasal morphology and airflow dynamics between humans and cynomolgus monkeys, and proposed the latter as a suitable surrogate for human inhalation studies. However, they noted the presence of minor variances in anatomy and airflow dynamics between the two species and urged caution in their interpretation. Mori et al. (127) proposed the numerical simulation of air-conditioning performance of six macaques (four *Macaca fuscata* and two *Macaca mulatta*) and a savanna monkey (*Chlorocebus aethiops* Linnaeus). They suggested that the evolutionary modifications in the nasal anatomy are independent of climate and atmospheric environment variations of the macaque’s habitat. In a further study (128), the authors compared the principles of air conditioning in humans, chimpanzees (*Pan troglodytes*), Japanese macaques (*M. fuscata*) and two rhesus macaques, (*M. mulatta*). Their findings indicated that the morphological variation of the nasal passage topology was weakly sensitive to the ambient atmosphere conditions; the high nasal cavity in humans seems to have developed by evolutionary facial reorganization in the divergence of Homo from the other hominin lineages. Bastir et al. (129) modeled airflow pressure, velocity, and temperature changes in six adult humans and six chimpanzees and analyzed 164 semi-landmarks of 10 humans and 10 chimpanzees with the aim of comparing 3D size and shape. They found significant differences in the internal 3D nasal airways.

Corley et al. (72) developed a high-resolution nose-lung model from scans of a rhesus monkey and simulated vapor deposition (acrolein) and subsequent pharmacokinetics (PK) in comparison to rat and human (Figure 10B). It was confirmed that the uptake of vapor in specific regions was influenced by various factors, including airway geometry, airflow rates, acrolein concentrations, metabolic rate, tissue thickness, and partition coefficient at the air-tissue interface. The study also predicted that the rats had the highest nasal extraction efficiency, followed by monkeys and then humans. Such information can be critical for understanding animal results and/or extrapolating animal data to humans. Given their close resemblance to humans, it is expected that non-human primate models of respiratory illnesses will remain critical in facilitating the translation of biomedical research for the betterment of human health, as well as in extrapolating laboratory data across different species (108).

### 3.8. Comparison of the nasal airway of land animals

In comparison to the nasal airways of a human (130–138) or monkey (139–141), the nasal airway architecture of macrosmatic animals such as dogs, rats, rabbits, and deer are much more complex (39, 72, 86–88). Notable differences can also be observed among non-primate animals. In dogs (*Canis familiaris*), the maxilloturbinate and ethmoturbinate exhibit a branching-type and scroll-type structure, respectively (39, 42). Out of the animals examined (dogs, rats, rabbits, and deer), the canine maxilloturbinate exhibits the most intricate structure. In the deer species (*Odocoileus virginianus*), the nose is characterized by a maxilloturbinate with a double-scroll-like structure and an ethmoturbinate with a branching-type structure. The deer’s ethmoturbinate exhibits the most intricate arrangement among the four species (86). Similar to the dog, the rabbit (*Oryctolagus cuniculus*) nose features a branching-type maxilloturbinate and a scroll-like ethmoturbinate (Figure 4). Nonetheless, the ethmoturbinate structure in rabbits seems significantly simpler than that of dogs. It may be too early to provide a definitive explanation for these anatomical distinctions, but it is possible that they have evolved as adaptations to external environments, given the distinct functions of maxilloturbinate (for respiration, air-conditioning, and cleaning) and ethmoturbinate (for olfaction).

Cat, rabbit, and deer have different sized and shaped ethmoidal turbinates, which reflect the different roles that their sense of smell plays in their respective habitats and behaviors. Cats have large and complex ethmoidal turbinates, which give them a greater ability to detect and analyze a wider range of odors. This increased surface area helps cats to identify specific scents, such as those of prey or predators, more easily. In comparison to cats, rabbits have smaller ethmoidal turbinates and a less developed sense of smell. This is because their main survival strategy is to rely on their sense of hearing and vision, as well as their agility and speed, to avoid predators. The smaller size of their ethmoidal turbinates reflects the lesser importance of their sense of smell in their survival and behavior.

By contrast, deer have larger ethmoidal turbinates than most other mammals, including cats. This is because their sense of smell plays a critical role in their survival, as they use it to detect predators, locate food, and communicate with other deer. The larger size of their ethmoidal turbinates reflects the importance of their sense of smell in their survival and behavior.

### 3.9. Phyllostomid bat nasal cavity morphology and olfactory flows

**Figure 11:**
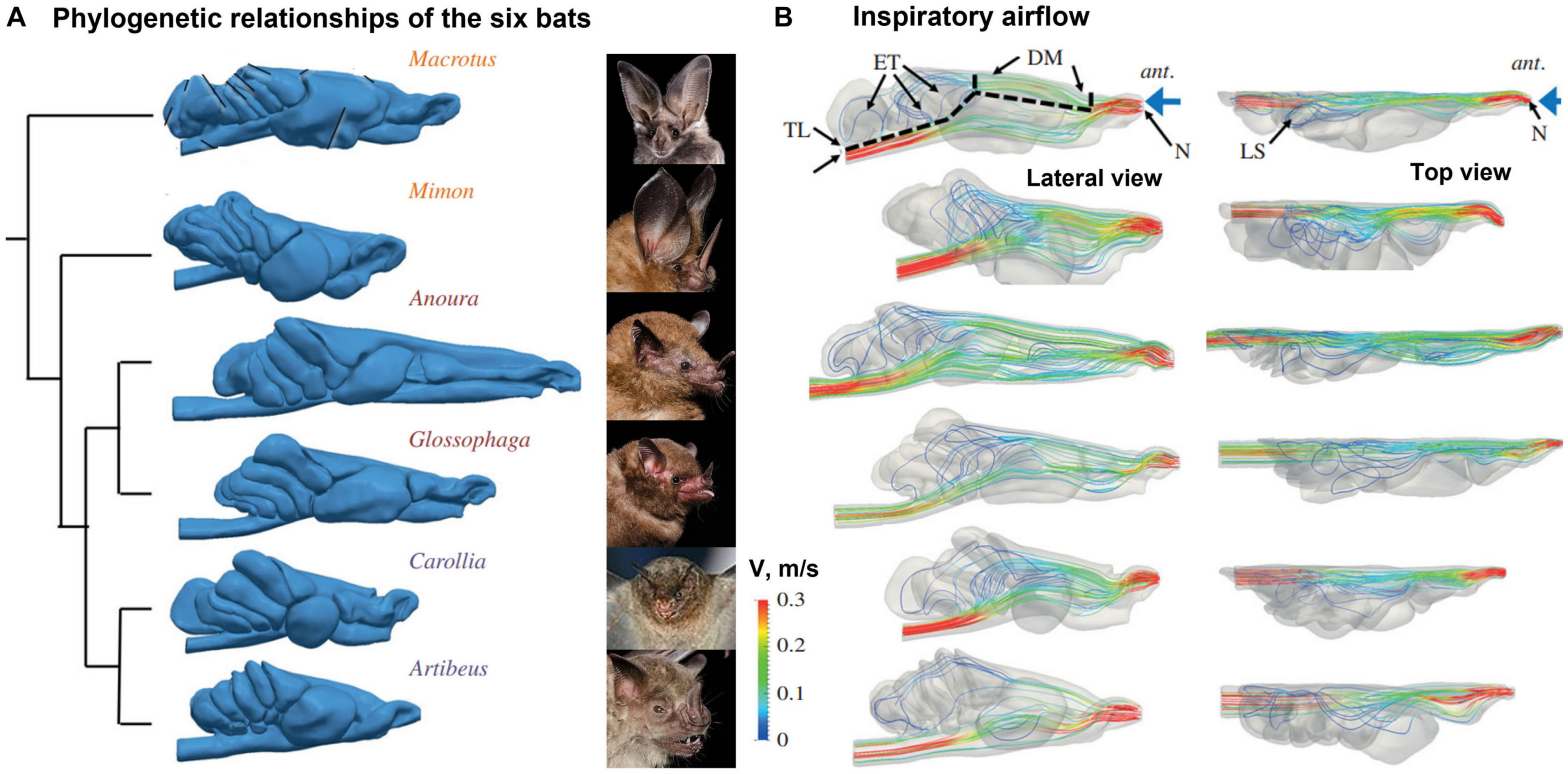
Phyllostomid bat nasal cavity morphology and olfactory flows (111): **(A)** phylogenetic relationships of the six bats, and **(B)** inspiratory flow patterns with lateral and top views.

Eiting et al. (111) examined the olfactory airflows among six bat species that have different nasal airway morphology and olfactory abilities (Figure 11A). They initially hypothesized that different morphologies were associated with different airflow patterns to the olfacory recess, which in turn could be explained by their dietary differences. Inhalation and exhalation airflow patterns and rates across six species were compared both qualitatively and quantitatively (Figure 11B). Contrary to the expectations, neither airflow patterns nor olfactory flow partitions were clearly different between species. The olfactory airflow remained consistent across various species, indicating that variations in the shape of the snout may be attributed to other functional requirements such as respiration and eating (111). On the other hand, they reported that the olfactory airflows could be improved by a larger olfactory recess during both inhalation and exhalation, and thus could be an important anatomical factor underlying a keen olfaction. As a blind pocket at the back of the nasal airway, the olfactory recess presumably sequestered the inspiratory airflows and allowed odors more recirculation time to be captured by the olfactory receptors.

### 3.10. Hammerhead shark: optimized hydrodynamics

The hammerhead shark has a unique nasal structure that sets it apart from other shark species. Its hammer-shaped head, or cephalofoil, has wide-spaced nostrils that are positioned on the ends of the head. These nostrils are located on the underside of the head, near the mouth, and are used to sample water for scents and tastes. The hammerhead shark’s broad head and wide-spaced nostrils enhance its ability to detect and locate odors, allowing it to locate prey more effectively. Numerous lamellae are present in the olfactory chamber, increasing the olfactory surface area. The hammerhead shark also has a higher concentration of sensory cells on the lamellae compared to other shark species, which further enhances its ability to detect scent.

**Figure 12:**
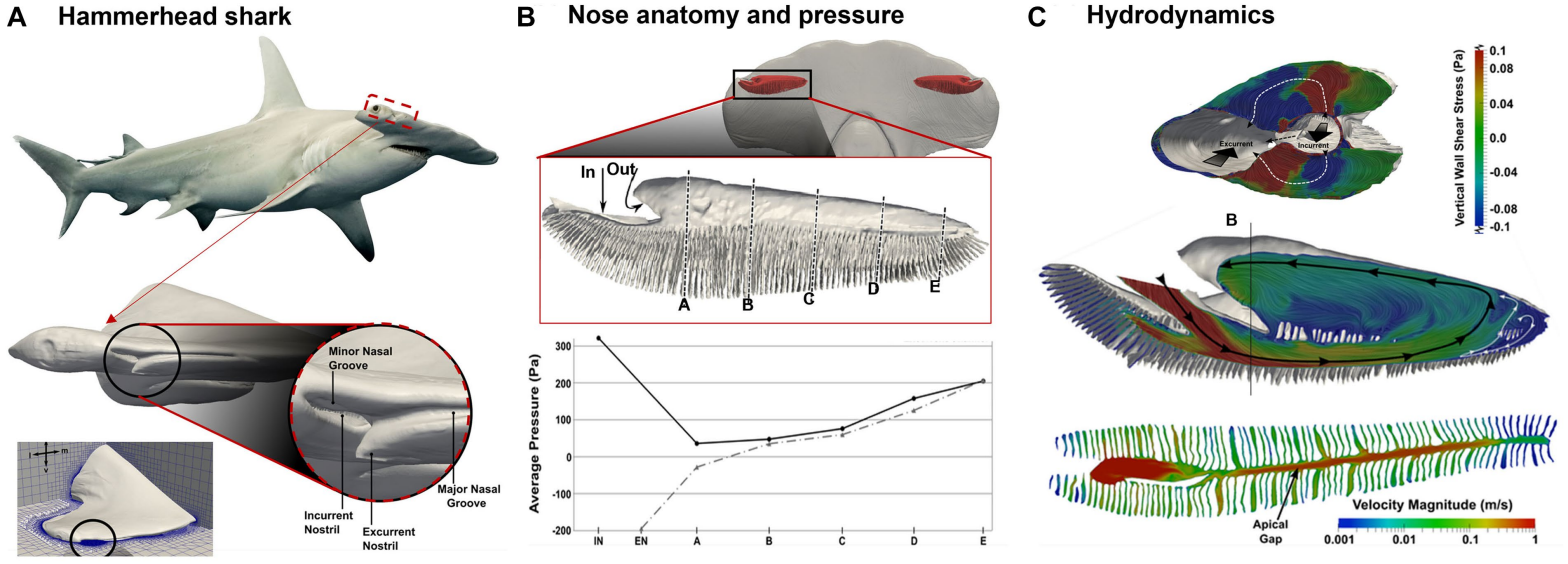
Hydrodynamics in the nasal region of a hammerhead shark (*Sphyrna tudes*) (142): **(A)** head and olfactory chamber, **(B)** pressure distribution along the incurrent and excurrent channels, and **(C)** internal flow patterns: surface-limited streamlines and velocity contours.

To better understand the hammerhead shark’s nasal anatomy and its implications in olfaction, Rygg et al. (142) developed a head model and olfactory chamber model based on MRI and micro-CT scans of a shark cadaver (Figure 12A). A simulation of the water flow in the reconstructed model reveals distinctive hydrodynamics of olfaction during swimming, as well as four functional structures regulating odor hydrodynamics for optimal odor detection in an aqueous environment. First, the olfactory chambers are located at the ends of the hammerhead (Figure 12B), and this wide lateral separation helps the localization of the odor source (tropotaxis) (143). Second, each olfactory chamber has an incurrent nostril and excurrent nostril (Figure 12A, lower panel), forming a uni-directional flow, as opposed to the bi-directional flows in land animals. The reason for this is that the incurrent and excurrent nostrils are positioned in areas of contrasting pressure (Figure 12A, lower panel). Specifically, the incurrent nostril can be found at the front edge of the cephalofoil, which is where the flow stagnation point generates the highest pressure. Meanwhile, the excurrent nostril is situated closer to the ventral side of the head, where the curvature of the nostril leads to accelerated flow and a resulting drop in pressure. Third, A broad nasal groove extends medially from the incurrent nostril along the front edge of the cephalofoil, directing a portion of the flow into the nostril (Figure 12A, lower panel). This mechanism enables the shark to sample a larger fluid volume and a wider spatial range. However, the nasal groove does not redirect all the flow into the incurrent nostril; a considerable amount is diverted away from the inlet. Consequently, the nasal groove’s configuration creates external flow patterns that increase the hydrodynamic range of the incurrent nostril while constraining the rate of incurrent olfactory flow. Fourth, flows within the sensory channels between olfactory lamellae can be regulated by the apical gap, which further varies the odor residence time (Figure 12C).

### 3.11. Fish and bird nasal airways: electric field and scent sensing

Chimaerids, also known as ghost sharks, are a group of ancient fish species known for their distinctive nose structure and electro-sensing ability (144). This organ consists of a series of jelly-filled canals that are sensitive to the slightest changes in electrical potential and vibrations. The jelly in the canals is a highly conductive material that amplifies the electrical signals, making them easier for the hagfish to detect. Additionally, the large number of ampullae distributed throughout the hagfish’s body gives it a highly sensitive and wide-ranging ability to detect both the electric field and pressure variation. As a result, the ampullae of Lorenzini can detect weak electrical signals or vibrations produced by the movement of other animals, including the muscle contractions and heartbeats of prey, which allows the chimaerid to locate food in dark or murky waters.

**Figure 13:**
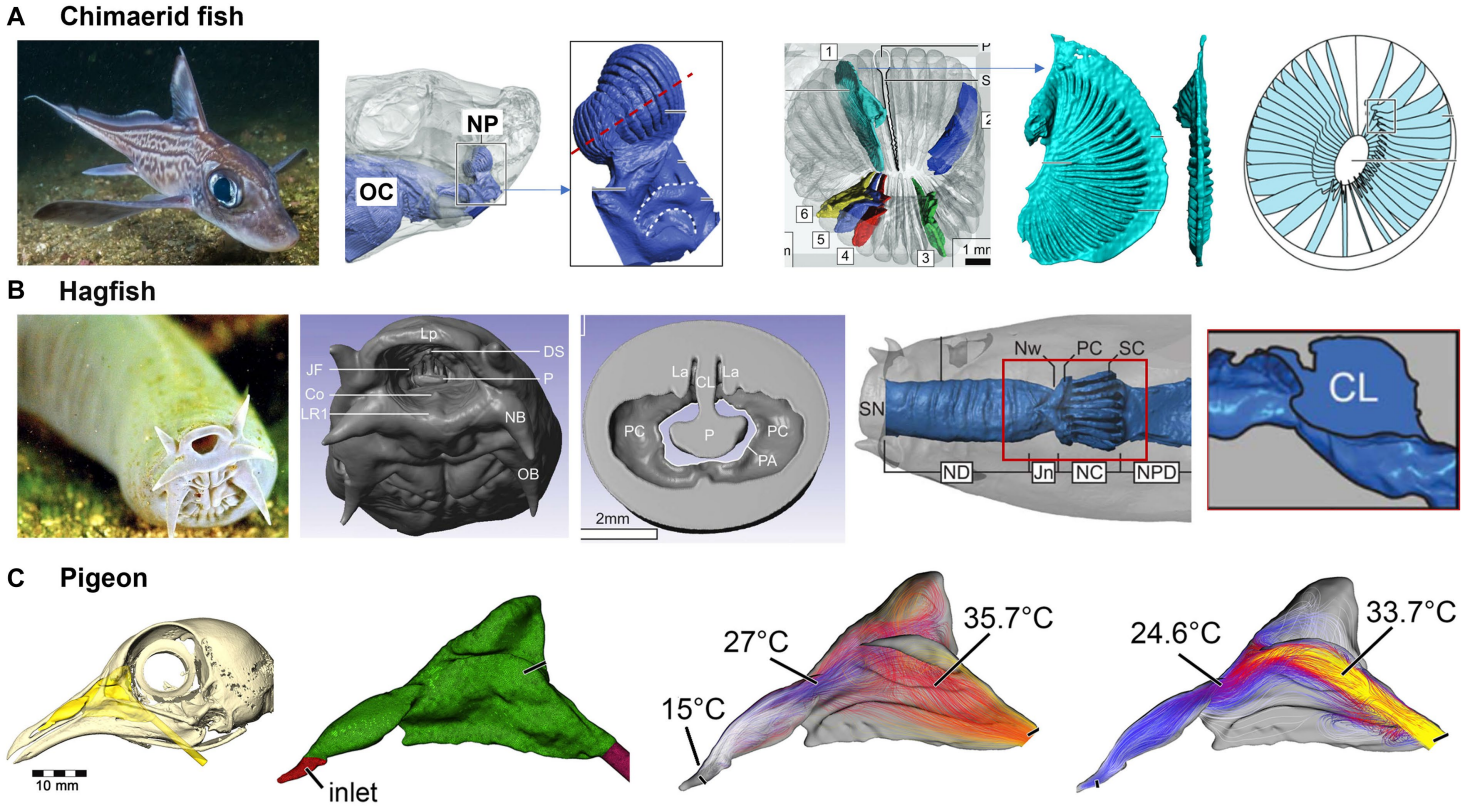
Fish and bird nasal airways: **(A)** functional nasal morphology of chimaerid fishes (145), **(B)** nasal passage of a hagfish (145), and **(C)** the nasal passage of a pigeon and airflow patterns (146).

Considering that signal transmission (either electrical, pressure, or chemical concentration) largely depends on the medium, Howard et al. (145) developed a model for water circulation in Chimaerid Fish’s nose based on both MRI scans for large structure and micro-CT scans for detailed anatomy such as the ampullae of Lorenzini. Different from land animals’ ethmoids, the Chimaerid Fish’s olfactory region contains an array of lamellae that formed from a radial arrangement around an elliptical support at the center (Figure 13A). Fluid circulation through the nasal cavity was simulated and several anatomical features were identified that segregated, distributed, and regulated flow in the nose. First, an incurrent channel connected the nasal chamber to the external environment and an excurrent channel connected the nasal chamber to the oral cavity, with both channels allowing water to flow through the nose. Second, non-sensory cilia line olfactory sensory channels and are mucus-propelling, suggesting that they protect cartilaginous fishes (sharks, rays, and chimeras) from harm. Thirdly, Chimaerid fishes’ nasal region shows adaptations to a benthic lifestyle, including secondary folding that increases the potential flat sensory surface area by 70% (145).

Hagfishs are eel-like creatures known for their exotic nose structure. Despite their primitive appearance, the scent detection system of hagfish is considered to be one of the most acute among all living organisms (147). They have a series of sensory tentacles covered in olfactory receptors, which can pick up the faintest of odors in the water. Holmos et al. (148) reconstructed a nasal passageway based on high-resolution MRI scans of an adult hagfish (Figure 13B). A long, broad passageway precedes the nasal chamber, which delays a response to odors by one or two seconds. The hagfish’s olfactory epithelium has a large surface area (~140 mm^2^), which maximizes odor accessibility. A slight expansion in the nasal chamber will cause inward flows and significantly enhance odor availability. Flow distribution across the olfactory region can be further facilitated by: (a) a convergent channel before the nasal chamber; (b) a partial nasal passageway obstruction by the central lamella; and (c) a slight inward tilt of the olfactory lamellae. The hagfish’s tentacles are constantly moving, which further facilitates the detection of scents from a great distance, even in the dark and murky conditions of the deep ocean where they live.

Pigeons have a highly efficient respiratory system that allows them to fly for long distances without getting tired. The air passages in their nose are designed to warm and moisten the air before reaching the lungs, which helps the pigeon conserve energy and maintain its stamina during flight. Whether pigeons have the ability to detect electric or magnetic fields is still a debate (149). This capability, or magnetoreception, has been found only in certain species of animals, such as migratory birds, sharks, and some species of turtles, which use the earth’s magnetic field for navigation (150). The effective thermoregulation of the pigeon’s nose has also been studied. Bouke et al. (146) simulated the airflow and heat exchange in a reconstructed nasal airway based on MRI scans of a pigeon head. Even with a less complex nasal morphology than other animals, the pigeon’s nose warmed the inhaled air by up to 22°C, bringing it close to body temperature before reaching the throat (Figure 13C). During exhalation, the temperature of inhaled air dropped from 38°C to 21.6°C before exiting the nostrils (i.e., a 16.4°C drop), rendering the pigeon nose a highly efficient heat exchanger in both warming cold air during inhalation and preserving heat during exhalation.

## 4. Animal models in COVID-19 research

**Figure 14:**
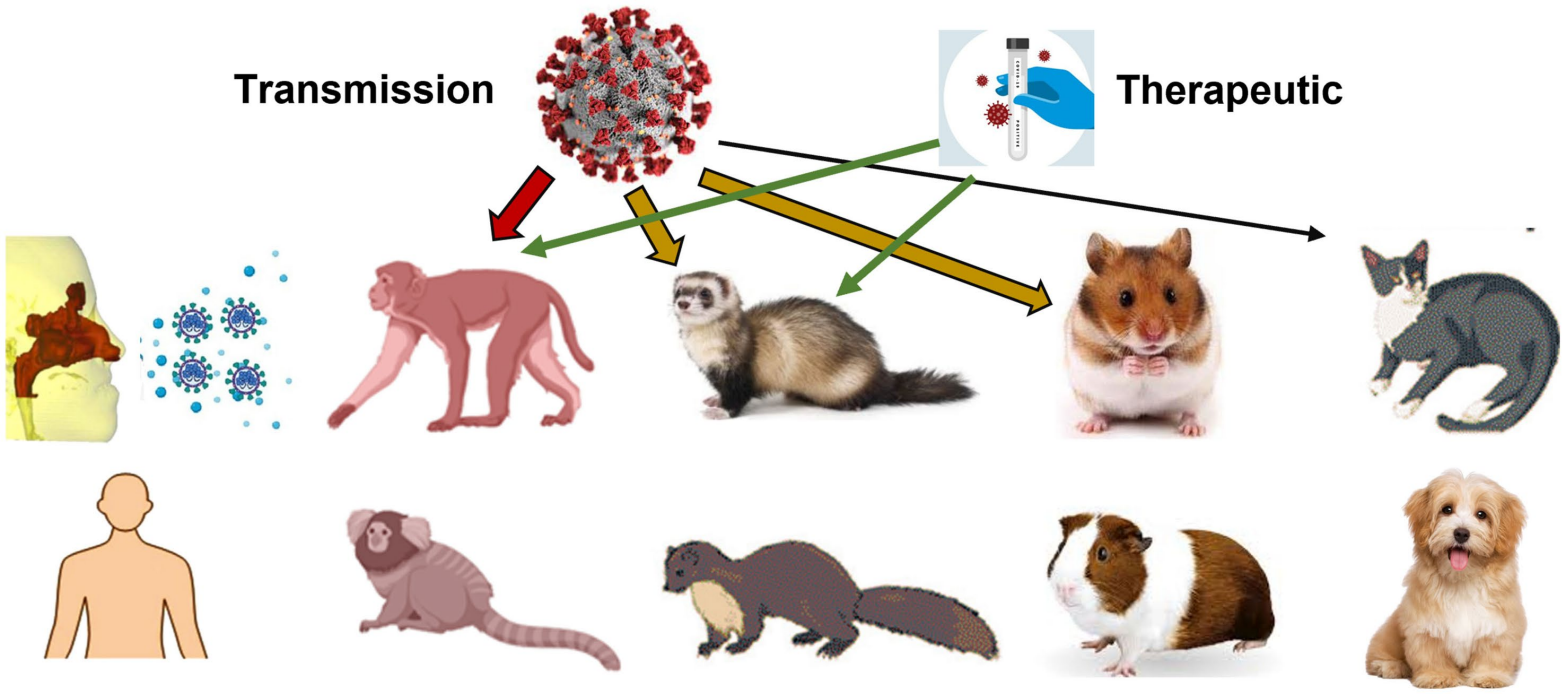
Animal models used in COVID research on virus pathogenesis, transmission, and efficacy of treatments and vaccines: primate, ferret, mink, hamster, guinea pig, cat, and dog.

Animal models have been an integral part of the COVID-19 research landscape since the emergence of the virus in late 2019 (Figure 14, first column). These models have enabled researchers to study various aspects of the virus, including pathogenesis, transmission, and efficacy of treatments and vaccines. Non-human primates, such as rhesus macaques, have been used as animal models to study the effectiveness of vaccines and treatments (Figure 14, second column). Non-human primates have a similar immune system to humans, which makes them valuable in evaluating the safety and efficacy of potential treatments and vaccines.

Ferrets, which are known for their similarities to human respiratory physiology, have also been used as animal models for COVID-19 research. Ferrets have been instrumental in studying the transmission of the virus and evaluating the efficacy of various treatments and vaccines (Figure 14, third column). These models have helped researchers understand the virus’s infectiousness, particularly through airborne transmission (151). To the best knowledge of the authors, there is no specific image-based airway model of the ferret. However, there have been several studies using ferrets as a model for respiratory diseases, such as asthma, and for understanding the mechanics of breathing. In these studies, images of ferret airways have been obtained using either computed tomography (CT) or magnetic resonance imaging (MRI) (152). These images have provided valuable information on the anatomy and function of ferret airways and have contributed to our understanding of respiratory physiology. However, to my knowledge, a dedicated image-based airway model of the ferret has not been established.

Hamsters have been used as a model to study the spread of the virus within a population (Figure 14, fourth column). These models have helped researchers understand the dynamics of transmission and the impact of various interventions on the spread of the virus (153). Dogs and cats have been studied in relation to COVID-19 (Figure 14, fifth column), mainly as potential hosts or carriers of the virus (154). They have also been used to study the effectiveness of vaccines and treatments for COVID-19 (155).

## 5. Limitations and future work

Despite the fact that micro-computed tomography (μ-CT) can currently be used to depict small nasal structures, it is not good at distinguishing olfactory or other mucosal types unless extra radio-opaque staining techniques are employed (156). MRI can better capture the mucosa than CT, but clinical MRI scanners often deficit in resolution (157). Despite being a thin layer (0.05–0.9 mm) (156), the presence or absence of the mucosa can noticeably affect the already-very-narrow airway passage, which will further affect the flow resistance, wall shear stress, and odor transport. Similarly, mucosa thickening or thinning that often occurs in the nose may also perceivably modify respiration and olfaction (158). One example is the nasal cycle, which exhibits spontaneous congestion and decongestion of the respiratory vascular mucosa (159). Perhaps due to the lack of measurement on nasal morphology evolution during a nasal cycle, no simulation studies have been reported on nasal cycle airflows, even though such results can have great implications on scent localization (direction and distance). It is also acknowledged that not all information is available for each species, and only relevant images that could be found in the current literature were presented. For instance, information on the nares, vestibules, airway passages, and cartilaginous structures was presented for the dog, rabbit, deer, sheep, pig, camel. But only information on image-based nasal airway geometry was presented for the rat, horse, and monkey.

This review provided an overview of the state-of-the-art characterization and modeling of the nasal anatomy and physiology of different animals. The advance in imaging techniques has enabled the visualization of the exquisitely intricate architectures of the nasal airway in 3D in great detail. The nasal structural similarities and uniqueness among animals provided valuable insights into both the keen sense of smell and the multifaceted adaptations to environments. The image-based modeling and simulation of animal respiration and olfaction further deepened our understanding of the myths underlying the olfactory acuity or humidity-thermal regulation efficiency in animals. Limitations included a limited number of high-quality animal nose imaging, the lack of physiological data in the majority of animals, and the incapacity of current imaging techniques to accurately capture the nasal mucosa. Further research in this field is likely to shed additional light on the complex interplay between nasal structure and function and may lead to new insights into the prevention and treatment of respiratory disorders and olfactory dysfunction.

## Author contributions

JX and MM contributed to conception and design of the study. JX, XS, and MM searched the database, selected relevant references, and conducted individual reviews. JX wrote the first draft of the manuscript. XS and MM wrote sections of the manuscript. All authors contributed to the article and approved the submitted version.

## Funding

The author MM is supported by grant PID2021-125731OB-C31 from the Spanish Ministry of Science and Innovation MCIN/AEI/10.13039/501100011033/and FEDER (“A way to build Europe”).

## Conflict of interest

The authors declare that the research was conducted in the absence of any commercial or financial relationships that could be construed as a potential conflict of interest.

## Publisher’s note

All claims expressed in this article are solely those of the authors and do not necessarily represent those of their affiliated organizations, or those of the publisher, the editors and the reviewers. Any product that may be evaluated in this article, or claim that may be made by its manufacturer, is not guaranteed or endorsed by the publisher.
